# Iptacopan in C5 blockade refractory atypical hemolytic uremic syndrome with associated Castleman’s disease: case report

**DOI:** 10.1186/s12882-025-04487-4

**Published:** 2025-10-24

**Authors:** Matthew D. Nguyen, Stefan Ciurea, Sheetal Desai, Arash Rezazadeh Kalebasty, Minh-Ha Tran, Caroline Gee, Mina Tadros, Vu Q. Nguyen, Samir Patel, Umut Selamet, Ramy Hanna

**Affiliations:** 1 Department of Medicine, University of California, Irvine School of Medicine, Irvine, CA USA; 2 Department of Medicine, University of Southern California/Los Angeles General Medical Center, Los Angeles, CA USA; 3 Department of Medicine, Division of Hematology/Oncology-Bone Marrow Transplant Unit, University of California-Irvine, Irvine, CA USA; 4 Department of Medicine, Division of Rheumatology, University of California-Irvine, Irvine, CA USA; 5 Department of Medicine, Division of Hematology/Oncology, University of California-Irvine, Irvine, CA USA; 6 Department of Pathology, Division of Transfusion Medicine, University of California-Irvine, Irvine, CA USA; 7 Department of Medicine, Division of Nephrology, Hypertension & Kidney Transplantation, University of California-Irvine, Irvine, CA USA; 8 Department of Medicine, Division of Oncology Dana Farber Cancer Institute, Harvard University, Boston, MA USA

**Keywords:** Atypical hemolytic uremic syndrome, Factor B blockade, Complement mediated thrombotic microangiopathy, Glomerular disorders, Membranous

## Abstract

**Background:**

Atypical Hemolytic Uremic Syndrome (aHUS) is a life-threatening disease related to mutations in the complement system. We report the first known case of the use of factor B inhibition in a 21-year-old male with complement-mediated thrombotic microangiopathy (CMTMA), later found to have Castleman’s disease as a mimicker of aHUS.

**Case presentation:**

Initially, the patient partially responded to eculizumab and then fully to ravulizumab. However, two years later, he experienced a flare that did not improve with ravulizumab redosing. An application for the compassionate use of Iptacopan was approved by the United States Food and Drug Administration, based on its success in C5 blockade Paroxysmal Nocturnal Hemoglobinuria. The patient’s blood counts recovered, his microangiopathic hemolytic anemia ceased, and his acute kidney injury resolved allowing cessation of renal replacement therapy. Human Anti-murine anti-drug antibodies were suspected as the cause of C5 blockade failure but were unconfirmed. Ten months later, the patient’s aHUS relapsed and he was concurrently diagnosed with Castleman’s syndrome, a mimicker of aHUS, prompting the initiation of dual combination therapy of C5 blockade inhibitor and Factor B inhibitor and siltuximab.

**Conclusions:**

This case marks the first use of Iptacopan for aHUS/CMTMA and the first use of dual combination therapy of eculizumab and Factor B Inhibition for patients with aHUS/CMTMA and Castleman’s disease in the United States at the University of California Irvine by our thrombotic microangiopathy assessment team.

**Clinical trial:**

N/A, this study is not a clinical trial.

## Introduction

Atypical Hemolytic Syndrome (aHUS) is a rare disease related to mutations in the alternative pathway of the complement system, as well as other genetic loci of proteins that interact with the complement system [1, 2]. Classically, aHUS presents with microangiopathic hemolytic anemia, thrombocytopenia, and acute renal failure, which can lead to kidney failure if unchecked in up to 50–60% of cases. There are many involved genes and heterogeneous presentations for this disease process [3, 4]. Defective control of the alternative pathway of the complement system at the endothelial level leads to initial thrombotic microangiopathy [1, 5]. The main causes of complement dysregulation are genetic or acquired mutations in complement factor H (CFH), anti-CFH antibody, membrane cofactor protein, complement factor I, and C3. Predisposing variants in CFH are the most common abnormalities seen in aHUS, accounting for 20–30% of cases. Variants in CFH associated with aHUS are mostly heterozygous and located in short consensus proteins that result in abnormal function but not quantitative deficiencies. Deletions in the genes encoding the five types of complement factor H related (CFHR) proteins are also highly implicated in aHUS. Acquired autoantibodies against CFH have also been identified in 5–20% of cases of aHUS and are highly associated with the genetic deletion of CFHR1 [2].

Differentiating between aHUS and other similar conditions, such as thrombotic thrombocytopenic purpura (TTP), vitamin B12 inborn errors, and Shiga toxin-induced hemolytic uremic syndrome (STEC-HUS), requires a process of elimination via stepwise algorithmic diagnosis. [6–8]. Regarding treatment of aHUS, plasma therapy (plasma infusion and exchange) had classically been used as first-line management. However, complement inhibition with anti-C5 blockers is now emerging as the gold standard treatment, typically with eculizumab or ravulizumab. Additional longer-acting agents and alternative blockers, such as C3, factor D, and factor B inhibitors, are in development [1, 2].

We describe a unique aHUS disease course with partial response to short-acting C5 blockade, complete response to long-acting C5 blockade which was then followed by acquired resistance to long-acting C5 blockade and finally response to rescue therapy with the novel Factor B inhibitor, Iptacopan. This was followed by a readmission while on Iptacopan due to a mimicker of aHUS, Castleman’s disease, with the patient now responding to dual therapy, consisting of C5 inhibition with Factor B inhibitor for his aHUS/complement-mediated thrombotic microangiopathy (CMTMA), and Siltuximab for Castleman’s disease.

## Case report part 1 - initial presentation

**Fig. 1:**
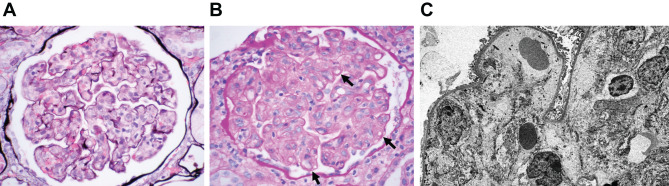
Renal biopsy findings. Mesangiolytic glomerulopathy, consistent with thrombotic microangiopathy. Acute tubular injury with mild interstitial inflammation and prominent tubular cell cytoplasmic vacuolization. Mild interstitial fibrosis with tubular atrophy. **A**) Jones methanamine silver stain with marked mesangiolysis with scattered entrapped red blood cells and red blood cell fragments. Fibrin thrombi were not present. **B**) Periodic acid-schiff stain with marked mesangiolysis and segmental double contours (shown by black arrows). **C**) Electron microscopy with mesangiolysis and marked subendothelial expansion by flocculent material

Our patient is a 19-year-old Pakistani male who presented to an institution outside of the University of California Irvine (UCI) with complaints of weakness, shortness of breath, and fatigue. Initial labs showed a serum creatinine of 5.4 mg/dL (ref. 0.7–1.3 mg/dL), uric acid of 8.8 mg/dL (ref. 4.4–7.6 mg/dL), hemoglobin of 6 g/L (ref. 13.5–16.9 g/dL), and platelets ranging between 84 and 116 thous/uL (ref. 150–400 thous/uL). Haptoglobin was not depleted at 231–237 mg/dL (ref. 44–215 mg/dL). Lactate dehydrogenase (LDH) was 437 U/L (ref. 96–199 U/L), coagulation studies showed a prothrombin (PT) time of 15.8 seconds (ref. 11.5–13.5 sec), international normalized ratio (INR) of 1.3 (ref. 0.9–1.10), and activated partial thromboplastin time (aPTT) of 41.4 seconds (ref. 24.3–34.9 sec). A PT/PTT mixing study was negative. Daily peripheral smear consistently shows “few” schistocytes. Initial D-Dimer was elevated to 16,620 ng/mL (ref. 215–500 ng/mL) and fibrinogen was also elevated at 528 mg/dl (ref. 200–393 mg/dL). C3 level was normal at 117 mg/dL (ref. 65–175 mg/dL). Renal biopsy done at outside hospital showed mesangiolytic glomerulopathy concurrent with thrombotic microangiopathy (TMA), acute tubular injury with mild interstitial inflammation and cytoplasmic vacuolization as the major findings (Fig. 1). After biopsy, the patient suffered bleeding followed by a page kidney complication and was transferred to UCI Medical Center.

Laboratory workup for rheumatologic diseases showed a 1:40 positive anti-nuclear antibody, but negative assays for anti-double stranded DNA, anti-ribonucleoprotein, and anti-smith antibody. A partial screen was positive for antiphospholipid antibody syndrome. The dilute Russell Viper Venom Time (DRVVT) was elevated, but anti-cardiolipin Immunoglobulin A, G, and M panels were negative.

Rasburicase, administered for suspected tumor lysis syndrome due to elevated uric acid, did not improve his symptoms. Lymph node biopsy and flow cytometry panels to rule out malignancy were negative for lymphoma or leukemia. Intramuscular vitamin B12 was empirically given due to a relatively low B12 levels (237 pg/mL, ref. 180–1241 pg/mL), although the presence of reticulocytosis and severe renal failure was consistent with complement-deficient TMA rather than severe B12 deficiency [9]. The absence of gastrointestinal symptoms provided low clinical suspicion for STEC-HUS. Our low suspicion for thrombotic thrombocytopenic purpura (TTP) was confirmed with a near-normal disintegrin and metalloproteinase thrombospondin motif 13, member 1 (ADAMTS13) level of 56%. At this time the diagnosis of atypical hemolytic uremic syndrome (aHUS) was considered. Following vaccination against Meningococcus types A and B, eculizumab was started.

**Fig. 2:**
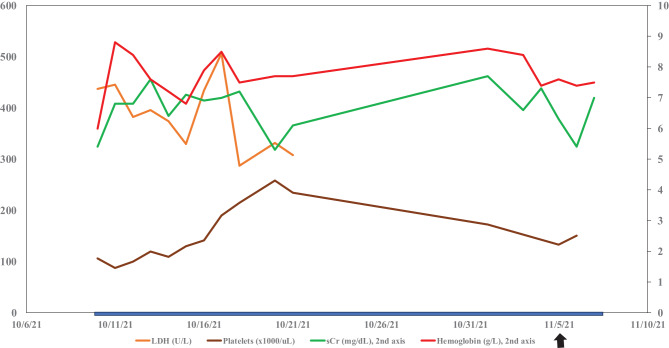
Lab trends initial presentation. LDH, lactate dehydrogenase; sCr, serum creatinine; Hgb, hemoglobin. Blue bar, patient on renal replacement therapy (continuous renal replacement therapy, or intermittent dialysis). Black arrow eculizumab started

**Fig. 3:**
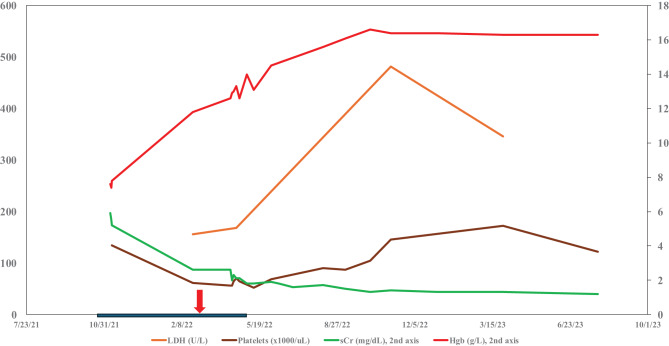
Lab Trends on C5 blockade. LDH, lactate dehydrogenase; sCr, serum creatinine; Hgb, hemoglobin. Blue bar, patient on renal replacement therapy (continuous renal replacement therapy, or intermittent dialysis). Red arrow ravulizumab started

The patient’s response lagged, and he remained on dialysis with partial hematologic response (platelets increased from 125 thous/mcL to 150 thous/mcL before dropping to 50 thous/mcL, hemoglobin remained between 7 and 8 g/dL) and renal response (creatinine decreased from 7.3 to 5.2 mg/dL) while on eculizumab. Overall creatinine clearance was < 15 mL/min given the patient’s small frame (about 50 kg). Given challenges with scheduling his eculizumab infusion, he was switched to ravulizumab. While on ravulizumab, he had rapid improvement of renal and hematologic parameters. Three months after the switch to ravulizumab, the patient was off dialysis with the best hematologic and renal remission numbers seen since presentation. Serum creatinine decreased from initial presentation of 5.4 mg/dL to the range of 1–1.4 mg/dL, platelets improved from its range of 10–50 thous/uL in the hospital to 100–150 thous/uL and hemoglobin improved from 6 g/dL to a range of 8–10 g/dL (Figs. 2 and 3).

## Case report part 2 - relapse while on C5 blockade

The patient remained free from the need of dialysis for 19 months but presented back to the emergency department (ED) with fatigue, 2 years after initial presentation to UCI. His serum creatinine had increased once again to 3.5 mg/dL. The patient continued to deteriorate with hemoglobin falling from the initial value of 11 g/L to 6.5 g/L and platelets falling from 56 to 9 thous/uL. Vitamin B12 levels remained high at > 1000 pg/mL, LDH remained in the normal range, schistocytes were observed rarely in the peripheral smear, aPTT was 58 sec, PT was 16.1 sec, and INR was 1.3. ADAMTS13 level was 55% and there was no clinical suspicion of STEC-HUS. C3 level was 197 mg/dL, up from 117 mg/dL 2 years prior. CH50 levels were checked (down from 45.6 U/mL to 25.4 U/mL) while receiving ravulizumab; however, this is not an accurate measurement of complement blockade when using ravulizumab.

The patient’s sudden cessation of an effective and complete TMA response was not previously seen in the adult literature with compliant patients. A possibility was the presence of human anti-murine antibodies since both C5 blocking agents on the market are humanized antibodies with murine sequences; however, a confirmatory antibody lab test was not performed. A repeat bone marrow biopsy was obtained showing no new significant changes other than chronic, nonspecific toxic insult due to previous aHUS. Genetic testing was performed with next generation sequencing and the Genome Reference Consortium Human Build 38 (GRCh38) as a reference group. Results were equivocal for complement disorder, and anti-complement factor H (anti-CFH) was negative. Notably, the patient was found to be heterozygous for the large CFHR1–CFHR3 deletion; the homozygous deletion is associated with Factor H auto-antibodies and aHUS. Additional genes associated with TMAs were sequenced and negative: ADAMT13, C2, C3, C3AR1, C4BPA, C4BPB, CD46 (MCP), CFB, CFH, CFHR1, CFHR2, CFHR3, CFHR4, CFHR5, CFI, DGKE, INF2, MMACHC, PLG, THBD, VTN, WT1.

In search for rescue therapies, C3, C5, Factor B, and Mannose binding lectin serine protease 2 (MASP-2) inhibitors were considered. Iptacopan emerged as a candidate due to its recent positive data in patients with paroxysmal nocturnal hemoglobinuria refractory to C5 blockade [10]. This agent was thus selected as a possible candidate for FDA emergency compassionate use authorization in an attempt to save the patient’s life. After discussion with the sponsor (Novartis pharmaceuticals), the US FDA, and the patient and family, a trial of Iptacopan was decided upon. Novartis pharmaceuticals provided Iptacopan with the FDAs approval for our case. Dosing for Iptacopan was 200 mg twice a day orally. Baseline C5b-9 just before Iptacopan was 234 ng/mL (ref. < 250 ng/mL).

**Fig. 4:**
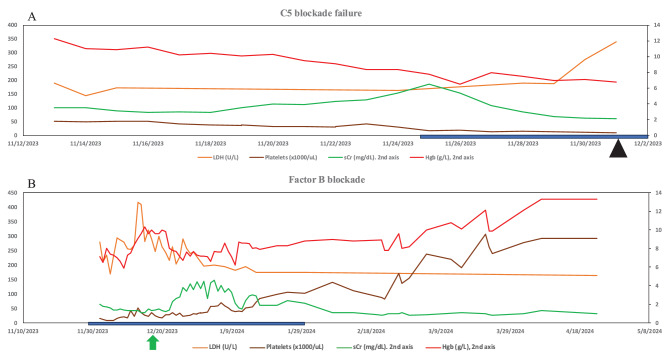
Lab trends relapse presentation and remission. LDH, lactate dehydrogenase; sCr, serum creatinine; Hgb, hemoglobin. Black triangle ravulizumab stopped. Green arrow iptacopan started. Blue bar, patient on renal replacement therapy (continuous renal replacement therapy, or intermittent dialysis)

Upon receiving Iptacopan, the patient’s cell counts at first slowly then rapidly improved, serum creatinine started dropping from a peak of 6.5 mg/dL down to the 2–3 mg/dL range, and within 2 weeks the patient started making urine. (Fig. 4) The patient had dialysis discontinued within 2 months, his hemoglobin had normalized (13 g/L), and for the first time ever his serum creatinine had normalized (0.8–1 mg/dL).

## Case report part 3 – relapse while on Iptacopan due to Castleman’s disease

10 months after initiation of Iptacopan treatment, the patient presented back to the ED with increasing abdominal distension and pain. He was afebrile. HgB on admission was 6.7 g/dL and platelets dropped to 35–40 thous/uL. Renal function had worsened with serum creatinine 4.8 mg/dL, BUN 33 mg/dL, and eGFR 15–18 mL/min/1.73 m^2^. Serum C5b-9 levels had risen to 250 ng/mL 8 months after Iptacopan initiation and 853.9 ng/mL at time of admission, reflecting lack of effective C5 blockade. A CT abdomen/pelvis was done showing no signs of active bleeding but did redemonstrate hepatosplenomegaly. A paracentesis was done showing straw-colored ascitic fluid negative for malignancy or infection. During this admission, the patient’s platelets continued to decrease to between 8 and 10 thous/uL, requiring frequent platelet transfusion. Genetic testing was repeated for concerns of molecular deficiency which came back equivocal. C3 level was elevated at 228 mg/dL and serum Factor H complement antigen was 35.9 mg/dL (ref. 18.5–40.8 mg/dL), with repeat negative anti-CFH. Initially, the TMA team was concerned that this readmission was due to a relapse in aHUS while on factor B blockade, as the patient had a positive response while on this medication. However, lymph node biopsy showed a mainly expanded medullary zone with reactive changes, including increased polyclonal plasma cells and vascularity. The biopsy was negative for malignancy and viral stains were negative. Bone marrow biopsy showed hypercellular marrow (90% cellularity) with moderate to severe reticulin fibrosis and moderately megakaryocytic hyperplasia and dysplasia, also negative for malignancy. Both biopsies were consistent with HHV-8 negative idiopathic multicentric Castleman disease vs TAFRO syndrome upon review by tumor board. After lengthy discussion and alternative considerations, the patient was diagnosed with TAFRO syndrome as the cause for this patient’s presentation mimicking a relapse in aHUS. 

**Fig. 5:**
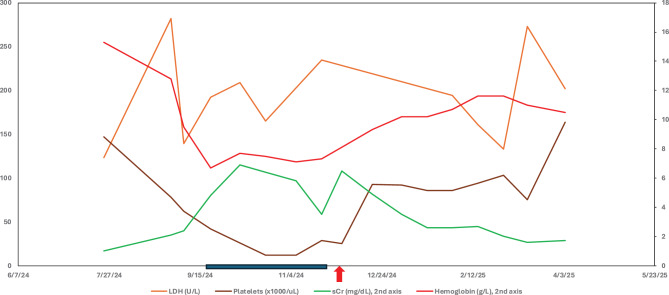
Lab trends with IL-6 and factor B inhibition LDH, lactate dehydrogenase; sCr, serum creatinine; Hgb, hemoglobin. Blue bar, patient hospitalized for relapse on Iptacopan. Red arrow, patient started on IL-6 blockade with Siltuximab for Castleman’s disease and factor B blockade (Iptacopan) for his atypical hemolytic uremic syndrome

TAFRO is a subtype of idiopathic multifocal Castleman disease consisting of thrombocytopenia, anasarca, fever, reticulin fibrosis, and organomegaly. The patient was started on dual combination therapy of C5 inhibitor (eculizumab) and Factor B blockade (Iptacopan) for his aHUS/CMTMA and then Siltuximab for his Castleman’s disease. Given his stronger prior response to Iptacopan, after 2 months his eculizumab was stopped and the Iptacopan was kept with the Siltuximab to address any CMTMA component given prior responses to complement blockade. The patient was transitioned to outpatient dialysis with close monitoring for severe thrombocytopenia requiring frequent platelet transfusion and anemia. Upon return to his outpatient visit 3 months later, his lab values had improved, with serum creatinine 2.4 mg/dL and stable anemia of hemoglobin 11.5 g/L and platelets 89 thous/uL (Fig. 5). The patient has been making urine daily, now requiring once weekly dialysis. He continues on Siltuximab and Iptacopan with close monitoring.

## Discussion

We present a patient with aHUS/CMTMA and a complement factor H-related protein 4 rare genetic variant who had a partial response to shorter acting C5 blockade, a more durable response to longer acting C5 blockade, and a relapse after nearly 19 months of dialysis-free renal and hematologic remission. This case report is significant and unique in three ways. To our knowledge, this is the first report of Iptacopan being used in the treatment of aHUS/CMTMA in addition to being the first report of its use resulting in suppression of aHUS before readmission due to a mimicker of aHUS, Castleman’s disease, while on Factor B inhibition. This case report is unique in that it is also, to our knowledge, the first reported case of dual combination therapy involving a C5 blockade (eculizumab) with a Factor B inhibitor (Iptacopan) in a patient with aHUS/CMTMA. Furthermore, this case also highlights the importance of exploring alternative mimickers of thrombotic microangiopathies and aHUS such as hematological pathologies such as Castleman’s disease.

AHUS is caused by a hyperactivation of the alternative complement pathway due to over activation of C3 convertases and subsequently loss of regulatory complement mechanisms [11]. First line treatment is through complement blockade [12]. However, failure in efficacy of complement cascade is multifactorial including genetic mutations, autoantibodies against the complement proteins, infectious, or pharmacokinetic nonadherence or inadequate dosing [2, 13]. In our case, this patient’s initial failure in eculizumab and success in ravulizumab was due to timely infusion due to the patient’s schedule rather than genetic, triggering factors, infectious or autoantibodies. After over one year being dialysis free on ravulizumab, this sudden failure in successful complement blockade has not previously been documented without any of the inciting events, warranting a search for a rescue therapy for this patient’s relapse.

Iptacopan is a proximal complement inhibitor that binds to factor B and inhibits the alternative complement pathway, with its use often being associated with IgA nephropathy [14]. Little knowledge is known about the efficacy of Iptacopan in relation to its efficacy in patients with aHUS refractory to C5 blocker therapy or dual combination therapy combining a factor B inhibitor and C5 blockade inhibitor. However current studies such as the Alternative Pathway Phase III to Evaluate LNP023 in aHUS (APPELHUS) trials are exploring the efficacy and safety of Iptacopan in patients with aHUS [14] Given that Iptacopan has not yet been approved for the treatment of aHUS, to our knowledge this is the first reported case of Iptacopan in refractory C5 blockade and Castleman’s disease as a mimicker of aHUS/CMTMA.

Idiopathic multicentric Castleman’s Disease (iMCD) is a rare lymphoproliferative disorder with features of thrombocytopenia, ascites, reticulin fibrosis, renal dysfunction, and organomegaly. TAFRO syndrome is one such subtype which has been noted to be a mimicker of thrombotic microangiopathies [15]. Renal complications for iMCD are fairly uncommon but have been seen to present in some patient cases [16]. It has been proposed that increased interleukin-6 (IL-6) levels, a marker associated with iMCD activity, may create a toxic environment for the renal microarchitecture that may induce thrombotic microangiopathies, similar to our patient’s renal biopsy [17, 18]. However, this patient’s extensive disease course is not due to strictly Castleman’s disease as the patient did respond to long-term C5 blockade and initially to Factor B inhibition. Treatment of multicentric Castleman’s disease is with Siltuximab which blocks IL-6, a prominent protein in the pathophysiology of iMCD progression [19]. However, we considered that C5 blockade and especially Factor B blockade may have directly addressed Castleman’s pathology via interactions with VEGF, a downstream target of IL-6 [20, 21].

In summary, this report suggests that Factor B blockade, and as an extension dual combination therapy of C5 inhibition and Factor B blockade, may be a robust option for rationale therapy in patients who have failed C5 blockade especially after initial success. Furthermore, this case emphasizes exploring and considering the many mimickers of TMA and the importance of a TMA assessment team to address common and rare presentations of this deadly group of diseases.

## Data Availability

No datasets were generated or analysed during the current study.
